# Tolvaptan Response in a Hyponatremic Newborn with Syndrome of Inappropriate Secretion of Antidiuretic Hormone

**DOI:** 10.1155/2021/9920817

**Published:** 2021-05-12

**Authors:** Cengiz Zeybek, Ali Dinç Bozat, Erhan Calisici, Ahmet Bolat, Belma Saygili Karagol

**Affiliations:** ^1^University of Health Sciences, Gulhane School of Medicine, Department of Pediatrics, Division of Pediatric Nephrology, Ankara, Turkey; ^2^University of Health Sciences, Gulhane School of Medicine, Department of Pediatrics, Division of Neonatology, Ankara, Turkey; ^3^University of Health Sciences, Gulhane School of Medicine, Department of Pediatrics, Ankara, Turkey

## Abstract

The use of tolvaptan to treat both euvolemic and hypervolemic hyponatremia has rapidly increased in recent years. However, data on its effects on children, especially newborns and infants, are limited. Here, we present a newborn who developed syndrome of inappropriate secretion of antidiuretic hormone following an intracranial hematoma drainage operation who was unresponsive to conventional treatments. The infant was successfully treated with tolvaptan, a competitive inhibitor of the vasopressin V2 receptor.

## 1. Introduction

Antidiuretic hormone (ADH) is produced in the hypothalamus and released from the posterior pituitary gland [1]. Normally, ADH is released under conditions such as hyperosmolality or hypotension, but pathological release occurs in euvolemic conditions such as syndrome of inappropriate secretion of antidiuretic hormone (SIADH) and in hypervolemic conditions such as congestive heart failure (CHF) and nephrotic syndrome (NS). SIADH can be triggered by pain, stress, numerous medications, and brain malformations; it also occurs in association with brain injury. The first-line treatment for SIADH is fluid restriction. Other treatment options include furosemide and sodium supplementation [2]. Data on the treatment outcomes of tolvaptan in children, where this drug specifically antagonizes AVPR2 receptors, are limited, especially for newborns. Here, we present a newborn infant who developed SIADH due to an intracranial hematoma operation and did not respond to conventional treatments but responded dramatically to tolvaptan treatment.

## 2. Case Report

Our female patient was born as a twin at 34 weeks and 5 days, via C-section, to a 19-year-old mother (G1P1). Her birth weight was 1,900 g (10–25^th^ percentile), and the head circumference was 32 cm (50–75^th^ percentile). She was born at another center, where she was followed for 5 days, diagnosed with of prematurity and oligohydramnios, and discharged without complications.

**Figure 1:**
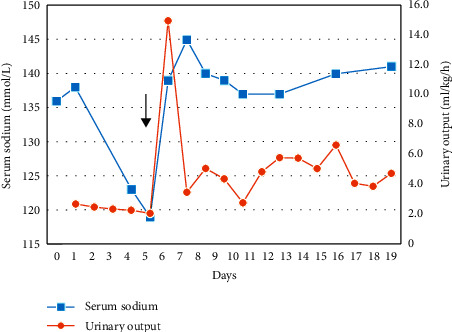
Serum sodium and urinary output before and after single-dose tolvaptan treatment. The black arrow indicates the day on which tolvaptan was administered.

She was admitted to the neonatal intensive care unit of our hospital with complaints of vomiting and neonatal convulsion on postnatal day 27. When hospitalized, her weight was 2,160 g, and blood gas analysis revealed that serum electrolytes (sodium: 136 mmol/L), glucose, calcium, magnesium, and other biochemical values were within normal limits. Transfontanel ultrasonography performed due to convulsions revealed a hematoma in the right lateral ventricle compressing the third ventricle and grade 3 intraventricular hemorrhage. The hematoma was drained, and an extraventricular drainage system was placed. After the operation, convulsions recurred, and the serum sodium level was 123 mmol/L. Convulsions were controlled with intravenous hypertonic sodium infusion, and fluid restriction was applied. During this period, hypotonicity was observed. Sodium supplementation was increased to a dose of 12 mmol/kg/d. Despite these efforts, normonatremia was not achieved, and the serum sodium level remained in the range 122–130 mmol/L. During this period, the urine density was 1019, and the urine sodium level was 120 mmol/L. Mild convulsions were observed during additional sodium replacement therapy on postnatal day 33; the serum sodium level during this period was 119 mmol/L, while the urine sodium was 154 mmol/L, serum osmolality was 270 mOsm/L, urine osmolality was 450 mOsm/L, and the plasma ADH level was 21.05 pmol/L (normal: <13). Based on these clinical findings, a diagnosis of SIADH was made. Thyroid and adrenal function, assessed by examining ACTH, aldosterone, and cortisol levels, along with thyroid function tests, were normal. Because the patient was hyponatremic despite fluid restriction and sodium supplementation, a single dose of 0.5 mg/kg/d tolvaptan was administered via a nasogastric tube, with consent from the parents, based on the doses in the literature published by Marx-Berger et al. (0.6 mg/kg/d in one case and 0.8 mg/kg/d in the other case) [3]. Hypertonic saline infusion continued with tolvaptan and then no more tolvaptan was required. The serum sodium level was 131, 139, and 152 mmol/L at 6, 12, and 18 hours, respectively; the urine output was 15 ml/kg/h. No side effects including liver or kidney dysfunction and neurological symptoms related to overcorrection of the sodium level were observed after tolvaptan treatment. On the contrary, at 24 hours after tolvaptan treatment, the patient's hypotonicity decreased. The next day, the serum sodium level spontaneously regressed to 140 mmol/L. The patient was discharged on postnatal day 46 with a serum sodium value of 140 mmol/L, urine output of 4.75 ml/kg/h, and stable neurological status and was scheduled to attend the outpatient clinic for follow-up. The patient's serum sodium and urinary output before and after single-dose tolvaptan treatment are shown in Figure 1.

## 3. Discussion

The main treatment modalities for SIADH are fluid restriction and sodium supplementation [4–6]. However, because the main nutritional source for newborns and infants is breast milk or formula, fluid restriction in these children results in inadequate caloric intake. Moreover, sodium supplementation can cause hypertension, which is difficult to control in these patients [3, 7]. Another treatment is oral urea; this induces osmotic effects but is sometimes poorly tolerated due to taste and is difficult to apply in newborns and infants. In recent years, the efficacy of the oral AVPR2 antagonist tolvaptan, a drug that specifically blocks AVPR2 receptors, has been demonstrated in euvolemic and hypervolemic adults [8]. However, data on the efficacy of the drug in pediatric patients, especially infants and newborns, are limited, and tolvaptan has not yet been licensed in Europe or the US for children. For this reason, liquid formulations of the drug are not available for young children.

In the research literature, the use of tolvaptan in newborns, infants, and children has been reported, mostly in cases with CHF [9–12]. Tolvaptan is an important drug in the treatment of CHF in adults who are resistant to diuretics [13–15]. Tolvaptan treatment was applied to 28 patients (mean age, 2 years; range: 1 month–18 years) by Regen et al. and to 34 patients (mean age, 49.1 ± 60.8 months; range: 2–202 months) by Higashi et al.; all of the patients were diuretic-resistant and had CHF [9, 10]. Normonatremia and an increase in urine output were observed in most cases within 3 days after single-dose tolvaptan in the first study, and in most cases after 1 month of tolvaptan therapy in the second study. Interestingly, in their logistic regression analysis, Higashi et al. showed that high urine osmolality was the only significant determinant of a good response to tolvaptan. In that study, urine osmolality was 439.2 ± 187.5 mOsm/L in the tolvaptan responder group and 237.3 ± 62.3 mOsm/L in the non-responder group. This is intuitive because the higher the urine osmolality is, the higher the serum ADH level will be and the more effective tolvaptan therapy becomes. In our case, urine osmolality was high before tolvaptan, at 450 mOsm/L. This may explain why our case responded so dramatically to tolvaptan therapy. Kerling et al. used multivariate regression analysis in a study of 25 newborns and infants with capillary leak syndrome after cardiac surgery [16]. They reported an increase in urinary output and mean blood pressure on the second day after tolvaptan therapy as criteria for a response to tolvaptan. Supporting this finding, in our case, urinary output increased up to 15 ml/kg/h after tolvaptan therapy.

Tolvaptan is also used in NS. Shimuzu et al. used tolvaptan not to induce normonatremia, but rather to treat severe edema in NS, as one of the factors implicated in edema pathogenesis in NS is the non-osmotic release of ADH [17]. Treatments such as albumin + diuretic and extracorporeal ultrafiltration were used to treat severe edema in a 16-year-old patient with steroid-resistant NS. The urine volume increased rapidly within 7 days after the initiation of tolvaptan therapy, and edema was dramatically improved.

Marx-Berger et al. applied tolvaptan therapy in two infants with SIADH aged 3 and 4 months (in one after pneumothorax and the other due to cranial midline defects) [3]. They reported a rapid response to the treatment in both cases, characterized by normonatremia. In the first case, as in our patient, tolvaptan was given at a dose of 0.8 mg/kg/d, which resulted in hypernatremia, but the hypernatremia spontaneously resolved by reducing the dose to 0.22 mg/kg/d the next day.

In adult patients with autosomal-dominant polycystic kidney disease (ADPKD), tolvaptan slowed the increase in kidney size and rate of glomerular filtration decline [18]. Magnetic resonance imaging of these patients revealed that the kidneys were reduced in size by 3.1%. Gilbert et al. started tolvaptan in a baby with severe hyponatremia and neonatal ADPKD at 32 days of age and continued this treatment until the age of 1 year [7]. In this study, kidney size was evaluated by ultrasonography, and neither growth nor recession of the kidney was detected. However, studies on the effects of tolvaptan in adolescent ADPKD patients are still ongoing [19].

In adults, potential adverse effects of tolvaptan include osmotic demyelination syndrome (ODS), resulting from rapid overcorrection of serum sodium levels, as well as liver injury, thirst, pollakiuria, and fatigue [20]. ODS is very infrequent in newborns and infants [21]. This rarity is probably due to the ongoing process of myelination, which begins in the fetal period and continues at least until the age of 2 years. This may explain why we did not encountered any neurological symptoms in our case, although the sodium value of 33 mmol/L increased with tolvaptan treatment within 24 hours.

In all of these pediatric studies, tolvaptan was used for months or years [7, 10, 22, 23]. Fortunately, no significant side effects have been reported with long-term treatment.

Unfortunately, vaptan is not available in liquid form. Tolvaptan is the only vaptan derivative used orally. According to the manufacturer's information, tolvaptan is insoluble in water, so crushing the tablet and mixing it with water is not advised due to the risk of incorrect dosage. However, we have observed good outcomes by crushing the drug and dissolving it in water in literature research.

In the literature, tolvaptan has been used at a dose of 0.05–1.3 mg/kg/d in children. Although we gave our patient tolvaptan at a dose of 0.5 mg/kg/d, which caused a nephrogenic diabetes insipidus-like syndrome the next day, based on the doses in the literature published by Marx-Berger et al., ideally treatment should start with the lowest possible dose, with subsequent dose adjustment according to urine output and serum sodium levels [3].

## 4. Conclusion

Tolvaptan can be used safely and effectively, even in neonates, as long as electrolyte levels and dehydration are carefully controlled in the neonatal intensive care unit.

## Data Availability

The data used to support the finding of this study are included within the article.
